# Accidents in the Country

**Published:** 1904-06-11

**Authors:** 


### ACCIDENTS IN THE COUNTRY.

THE following letter from Sir Henry Burdett appeared in the *Times* of Saturday last: On Whit Monday an accident happened to me in the country which might fall to the lot of any one at any time. I was driving in a wagonette along a country lane, when, on turning into a main road, a motor-car appeared, coming towards us, being about a quarter of a mile off. The horse became restive and then bolted in the direction of the motor-car, when the coachman succeeded in turning the horse off the road on to some rough grass to the left of it, near a brickfield. This excited the animal to greater speed, and unfortunately there were some heaps left by the roadside which the coachman could not avoid, which resulted in the carriage being upset violently on its left side and then turned turtle. There were four occupants of the carriage, one of whom, a lady, was uninjured; Canon Lawson, who had a throat wound and fractured his left arm; the coachman, who was severely bruised and cut about the head; and myself. I occupied a low seat by the side of the driver which gave me no purchase, and when the horse bolted and the carriage came into collision with the heap I was shot with great violence ahead of the carriage. When the vehicle turned over I received several violent blows, was spun along like a teetotum, and with two final blows over the region of the heart escaped without further maltreatment. I became unconscious, and it subsequently transpired that, in addition to concussion, I had received several fractures, the worst being that of the left shoulder joint, involving all the bones entering into that joint. Almost every part of my body was bruised or lacerated, but, fortunately, my head and right arm were practically uninjured, though both were bruised.

I give these details in order to enforce the point which I wish to make. The condition to which I was reduced gave me the most excruciating pain, for there was only one small place on the right side where I could suffer pressure at all, on which I have had to lie. My condition, which may be the condition of anyone to-morrow in a country district who meets with a similar accident, will best be realised when I state that for nine days and nights it was only possible for me, owing to the pain, to obtain in the whole seven hours' natural sleep, and they were due to the provision of certain appliances which I obtained from London. The arrival of these appliances at once reduced my sufferings by 80 per cent.

Now severe accidents, involving great pain and suffering, are constantly happening in the country—in the hunting field, to cyclists, and from motor-car and carriage accidents. No ordinary house, and no hotel or inn, as at present constituted, contains any special provision for the comfort of cases of this kind, a fact which calls for a speedy remedy. I venture to suggest, therefore, that every hunt committee and the various cycle and motor clubs should take this matter in hand and provide fracture beds with every modern appliance for the comfort of sufferers from severe accidents, which could be obtained whenever needed in the districts where the accidents occur. The amount involved in making such provision is relatively small, but the saving in human suffering and in the risks to life would be great. I have now organised one complete accident equipment of the kind for my own comfort, which has brought me sleep when sleep was denied me, and has so supplemented the zealous skill of my medical attendants and nurses that there is every prospect of my making a perfectly good recovery within a reasonable time. I shall be glad to co-operate with any one with the view to the provision of such an accident equipment who may entertain the suggestion I here make and determine to provide it in the district where they may live in the country.

Having almost miraculously escaped death, I desire to remind my colleagues and friends in the Metropolitan Press, who have so generously co-operated with me for 30 years at least in the cause of the hospitals, that Sunday, June 12, is Hospital Sunday. It has been my privilege to supply them each year with figures and materials in order to bring this fact with telling force to the notice of the public during each weekday commencing Monday, June 6th next. I have, with as much self-command as I could muster, done my best this year, but am conscious of necessary imperfections and possibly diminished force in the appeal I have somewhat painfully prepared. May I therefore through your courtesy appeal to every editor and writer in the Metropolitan Press to help me with their sympathy by making a special effort themselves to arouse the public this year to the claims of the Hospital Sunday Fund? They will so secure that the hospitals shall gain rather than lose by my enforced absence from the army of workers and preachers who will unite to help the hospitals throughout next week and on Hospital Sunday, June 12th, 1904.

